# FACS-Based Functional Protein Screening via Microfluidic Co-encapsulation of Yeast Secretor and Mammalian Reporter Cells

**DOI:** 10.1038/s41598-020-66927-5

**Published:** 2020-06-23

**Authors:** Desislava Yanakieva, Adrian Elter, Jens Bratsch, Karlheinz Friedrich, Stefan Becker, Harald Kolmar

**Affiliations:** 1 Institute for Organic Chemistry and Biochemistry, Technical University of Darmstadt, Alarich-Weiss-Strasse 4, D-64287 Darmstadt, Germany; 2 Institute for Biochemistry II, University Hospital Jena, 07743 Jena, Germany; 3 Protein Engineering and Antibody Technologies, Merck Healthcare KGaA, Frankfurter Straße 250, D-64293 Darmstadt, Germany; 4 Merck Lab @ TU Darmstadt, Alarich-Weiss-Strasse 8, D-64287 Darmstadt, Germany

**Keywords:** Biochemistry, Immunology, Biotechnology

## Abstract

In this study, we present a straightforward approach for functional cell-based screening by co-encapsulation of secretor yeast cells and reporter mammalian cells in millions of individual agarose-containing microdroplets. Our system is compatible with ultra-high-throughput selection utilizing standard fluorescence-activated cell sorters (FACS) without need of extensive adaptation and optimization. In a model study we co-encapsulated murine interleukin 3 (mIL-3)-secreting *S. cerevisiae* cells with murine Ba/F3 reporter cells, which express green fluorescent protein (GFP) upon stimulation with mIL-3, and could observe specific and robust induction of fluorescence signal compared to a control with yeast cells secreting a non-functional mIL-3 mutant. We demonstrate the successful enrichment of activating mIL-3 wt-secreting yeast cells from a 1:10,000 dilution in cells expressing the inactive cytokine variant by two consecutive cycles of co-encapsulation and FACS. This indicates the suitability of the presented strategy for functional screening of high-diversity yeast-based libraries and demonstrates its potential for the efficient isolation of clones secreting bioactive recombinant proteins.

## Introduction

The importance of biopharmaceuticals (biologics) in medicine is increasing at a fast pace and the biologics market is predicted to reach nearly 400 billion USD/year by 2025^1^. Frequently applied biologics comprise substances such as cytokines, monoclonal antibodies, hormones, soluble receptors, recombinant DNAs, enzymes, and synthetic vaccines.

While biologics in targeted therapies often demonstrate remarkable safety and specificity, especially in case of autoimmune diseases^2^ and cancer^3^, the discovery of novel molecules and the necessary functional validation still represent a bottleneck in the development of novel biopharmaceuticals. To overcome these shortcomings, powerful display technologies such as phage display^4^ and yeast surface display^5^ have been developed which allow for the isolation of specific high-affinity molecules and respective genes from complex variant libraries. However, identified binders frequently show poor physiological activity in a biological context. Thus, extensive secondary functional screens are necessary for identification of hit molecules with the desired functional activity. Furthermore, those screens require elaborate subcloning of the surface-displayed hits into soluble expression formats and outcoming clones frequently demand further optimization of physicochemical and pharmacokinetic properties^6^.

Consequently, implementing functional assays and phenotypic screens in an earlier selection phase appears highly beneficial for the discovery of new potent biologic drugs or even first-in-class medicines with novel molecular mechanisms of action^7^. In this context, a major limitation is represented by the relatively low throughput of classical phenotypic screens, falling far behind the performance of high-diversity library-based approaches resting on affinity-driven selection protocols^8^.

Complex protein-protein interactions^9^, cell internalization^10^, receptor clustering^11^, or effector cell interactions (CDC, ADCC)^12^ underlie the modes of action of most biopharmaceuticals. Functional assays for novel biologics are mainly based on mammalian cell culture and individual, time- and cost-intensive activity screening of soluble candidate molecules. Miniaturized microplate-based assay formats can considerably increase the throughput, but necessitate more and more complex automation systems for low-volume liquid dispensing and sample analysis^13^.

Technical progress in the field of microfluidics^14^ has opened new possibilities for cell-based drug screening. In recent years, methodologies in continuous-flow microfluidics for a simultaneous test of compounds on different cell types, 3D cell culture on chips for more accurate simulation of tissue structures and physiological conditions, and droplet microfluidics for fast generation of millions of individual microreactors with volume in the picolitre range, have emerged^15^. In droplet-based microfluidics, cells and reactants are compartmentalized in small volume aqueous droplets, utilizing a microfluidic chip with distinct channels for the aqueous phase(s) and for the continuous phase - inert carrier fluid (oil)^16^. This technology opens the door to drug screening on a single-cell level, allowing for a drastic increase in throughput and, most importantly, for genotype-phenotype coupling. Compartmentalization of individual cells in distinct droplets entraps all secreted molecules and analytes, preventing interference with other cells and their products^17^.

As yet, screening of droplets and direct sorting of water-in-oil (w/o) emulsions on custom-made FADS (fluorescence-activated droplet sorting) devices^18^ require sophisticated operation skills. Moreover, FADS devices reported to date have relatively low sorting rates and limited variability in fluorescence detection compared to commercially available multichannel fluorescence-activated cell sorters (FACS)^19^. Since commercial FACS devices work with aqueous buffers as sheath fluid, the advantages of these instruments over FADS has so far not been fully exploited to analyze and process w/o emulsions. One strategy for combining droplet-based compartmentalization with FACS is the generation of double emulsions (water-in-oil-in-water or w/o/w), where the primary (w/o) emulsion is injected into a hydrophilic microfluidic chip containing an aqueous continuous phase (buffer). This procedure results in droplets consisting of an aqueous core surrounded by a thin oil shell, which in turn are dispersed in an aqueous solution and has successfully been implemented for the directed evolution of enzymes^20–22^. A drawback of this technique which makes it widely incompatible with mammalian cell-based readouts, is that the second emulsification step necessitates the use of detergents for the stabilization of the oil layer^23,24^. An alternative approach to FACS-based sorting of compartmentalized cells lies in the addition of hydrogel-forming polymers (agarose, alginate, Matrigel, etc.) to the cell-containing aqueous solution prior to encapsulation. This procedure does not impair droplet generation and moreover, retains cell viability and growth since hydrogel structures mimic functions of the extracellular matrix. For this reason, cell-laden hydrogel beads have extensively been used for the cultivation of mammalian cells in three-dimensional matrices and the generation of multicellular spheroids^25–27^. Importantly, it was demonstrated that after hydrogel solidification, microbeads can be readily recovered, transferred to an aqueous buffer and further processed by FACS^28^. The potential of this technique for the functional selection of recombinant proteins from variant collections by genotype-phenotype coupling has not yet been exploited.

**Figure 1:**
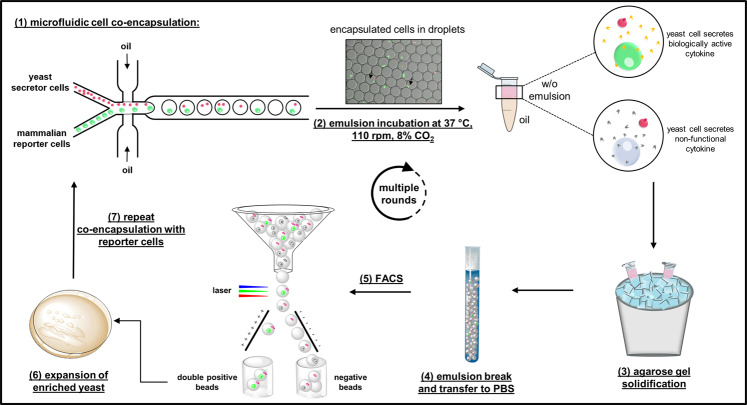
Workflow of the high-throughput functional screening procedure: **(1)** Microfluidic cell co-encapsulation of yeast secretor cells and mammalian reporter cells. Flow-focusing microfluidic chip with two aqueous channels enables the co-encapsulation of the two cell types in monodispersed aqueous droplets. Intracellular mCherry fluorescence of the yeast cells and GFP fluorescence of the reporter cells enable analysis of the cell distribution in the droplets directly after encapsulation – black arrows indicate droplets containing both cell types. **(2)** Cultivation of the encapsulated cells in emulsion overnight leads to loss/reduction of GFP fluorescence in the case of yeast cells secreting non-functional cytokines, while only the droplets containing yeast cells secreting biologically active molecules exhibit strong GFP fluorescence. **(3)** Agarose in the droplets is solidified by incubation on ice to form hydrogel microbeads. **(4)** After emulsion breaking the agarose microbeads are transferred to aqueous buffer (PBS). **(5)** Sorting of double-fluorescent agarose microbeads by FACS. **(6)** Sorted hydrogel microbeads are plated on selective agar for expansion of yeast cells. **(7)** Yeast clones are harvested and screening cycle is repeated.

Here we report a novel approach for ultra-high-throughput functional screening and selection of active protein biologicals based on co-encapsulation of individual *S. cerevisiae* secretor and mammalian reporter cells in agarose-containing microdroplets followed by fluorescence-activated cell sorting (FACS)(Fig. 1). As a model experiment, we demonstrate the FACS-based selection of yeast cells secreting functional murine interleukin-3 (mIL-3) by pairwise co-encapsulation with murine reporter cells which respond to mIL-3 stimulation by expression of green fluorescent protein (GFP). By two rounds of co-encapsulation and fluorescence activated sorting using a standard FACS instrument, mIL-3 secreting yeast cells could be rapidly and efficiently enriched and selected from a 1:10,000 dilution with yeast cells expressing an inactive mIL-3 variant.

This generic approach may also be amenable to the functional ultra-high-throughput screening of other biologics beyond cytokines, where a protein variant is displayed or secreted by yeast, while an activity-dependent fluorescence readout of live mammalian reporter cells allows for their isolation and identification.

## Results

### Murine interleukin-3-dependent activation of a reporter cell line

**Figure 2:**
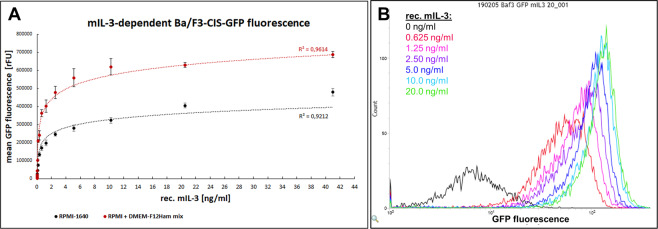
Reporter cell GFP fluorescence in dependency to the mIL-3 concentration. (**A**) Mean GFP fluorescence determined by flow cytometry after overnight incubation of Ba/F3-CIS-d2EGFP reporter cells in a 96 well plate with a dilution row (2.5 pg/mL – 41 ng/mL) of recombinant mIL-3 in standard cultivation medium (RPMI-1640) or a medium mix (50% RPMI-1640 + 50% DMEM-F12 Ham + 2% w/v galactose). GFP signal was normalized to the negative control (reporter cells incubated without mIL-3). (**B**) Flow cytometry histogram of reporter cells activated with different concentrations of rec. mIL-3 obtained on BD Influx cell sorter.

A murine Ba/F3 reporter cell line, expressing green fluorescent protein (GFP) upon stimulation with murine interleukin-3 (mIL-3) was constructed for a proof-of-concept study. Interleukin-3 is an important regulator of hematopoiesis and supports the growth of pluripotent stem cells and progenitors, as well as functional activity of some fully differentiated cells^29^. The responsiveness of the mIL-3-inducible Ba/F3-CIS-d2EGFP reporter cell line in relation to the mIL-3 concentration was examined and revealed a dose-dependent increase in the GFP fluorescence intensity upon stimulation with recombinant mIL-3 with concentrations in the range of 0.01–40 ng/mL (Fig. 2A, black curve). Furthermore, flow cytometry analysis confirmed cell population separation of the non-activated (0 ng/mL mIL-3) and the weakly-activated (0.625 ng/mL mIL-3) reporter cells (Fig. 2B), which is an essential prerequisite for efficient positive selection utilizing FACS.

### Generation of a non-functional mIL-3 mutant

**Figure 3:**
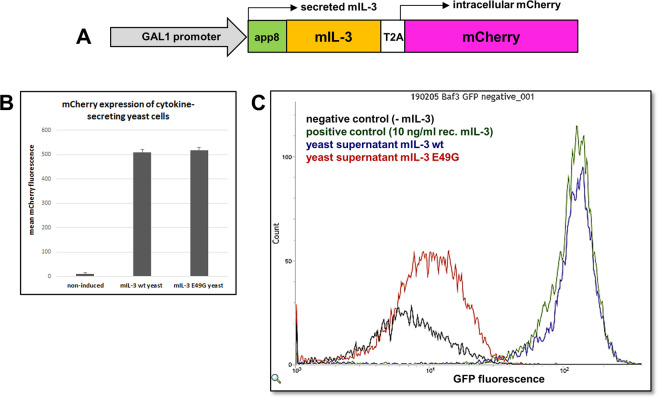
Analysis of mIL-3-secretion from *S. cerevisiae* cells. (**A**) Gene construct for the expression and secretion of mIL-3 in *S. cerevisiae*. Galactose-induced transcription (GAL1 promoter) leads to generation of a single mRNA transcript comprised of the transcripts of mIL-3 and a red fluorescent protein (mCherry). Ribosomal skipping peptide (T2A) between the two genes facilitates the translation of two separate proteins: mIL-3 with N-terminal secretion signal (app8) is secreted in the culture medium, while mCherry remains trapped intracellularly and serves as an expression control. (**B**) Mean mCherry fluorescence of yeast cells before and after induction with galactose. (**C)** Flow cytometry histogram of Ba/F3-CIS-d2EGFP reporter cells incubated with yeast supernatant after expression and secretion of mIL-3 wt or mIL-3 E49G for 24 h at 30 °C. As positive and negative controls, reporter cells were cultured in standard (RPMI-1640) medium with 10 ng/mL rec. mIL-3 or without mIL-3, respectively.

For verification of the functional screen approach, a non-activating mIL-3 variant with minimal sequence alterations (single amino acid substitution) was generated. Klein *et al*. studied in 1997 the receptor-binding site of human IL-3. In this study, one hIL-3 mutant (E22G) showed strong (>300 fold) reduction in growth-promoting activity, while retention of relative binding to the α receptor subunit of human IL-3 receptor^30^. Through a structural comparison of the human and murine IL-3 proteins (Supplementary Fig. S2), we identified the corresponding amino acid position in the mIL-3 ortholog, resulting in a non-activating mIL-3 mutant, E49G (Fig. 3C).

### Activation of Ba/F3-CIS-d2EGFP reporter cells using mIL-3 containing yeast supernatant

To enable detection of agarose microbeads containing an actively secreting yeast cell, a gene construct combining mIL-3 and mCherry genes under the control of the same GAL1 promoter was designed (Fig. 3A). Ribosomal skipping (T2A peptide) results in the secretion of the mIL-3 protein, carrying a secretion signal (app8^31^) (Supplementary Table S1). Simultaneously, mCherry fluorescent protein accumulates intracellularly in a correlation to the expression rate of the cytokine, as demonstrated by Grzeschik *et al*.^32^, and thus serves as an expression control.

In order to verify the secretion of functional wt cytokine (mIL-3 wt), Ba/F3-CIS-d2EGFP reporter cells were incubated with yeast culture supernatant after yeast induction and cytokine expression for 24 h (Supplementary Fig. S3). As a result, reporter cells incubated with the culture supernatant of the mIL-3 wt-secreting yeast exhibited strong GFP fluorescence, while yeast supernatant containing the mutated mIL-3 variant (E49G) induced GFP expression levels, comparable to the negative control-reporter cells depleted from mIL-3 (Fig. 3C). Since fluorescence levels of the intracellular mCherry expression controls were identical for both mIL-3 variants (Fig. 3B), and the discrepancy in the yeast culture densities was negligible, this result indicates successful cytokine secretion and accumulation in the yeast supernatant and at the same time verifies our assumption, that the single amino acid substitution E49G leads to strongly diminished reporter cell activation.

### Growth of *S. cerevisiae* EBY100 in different mammalian culture media

**Figure 4:**
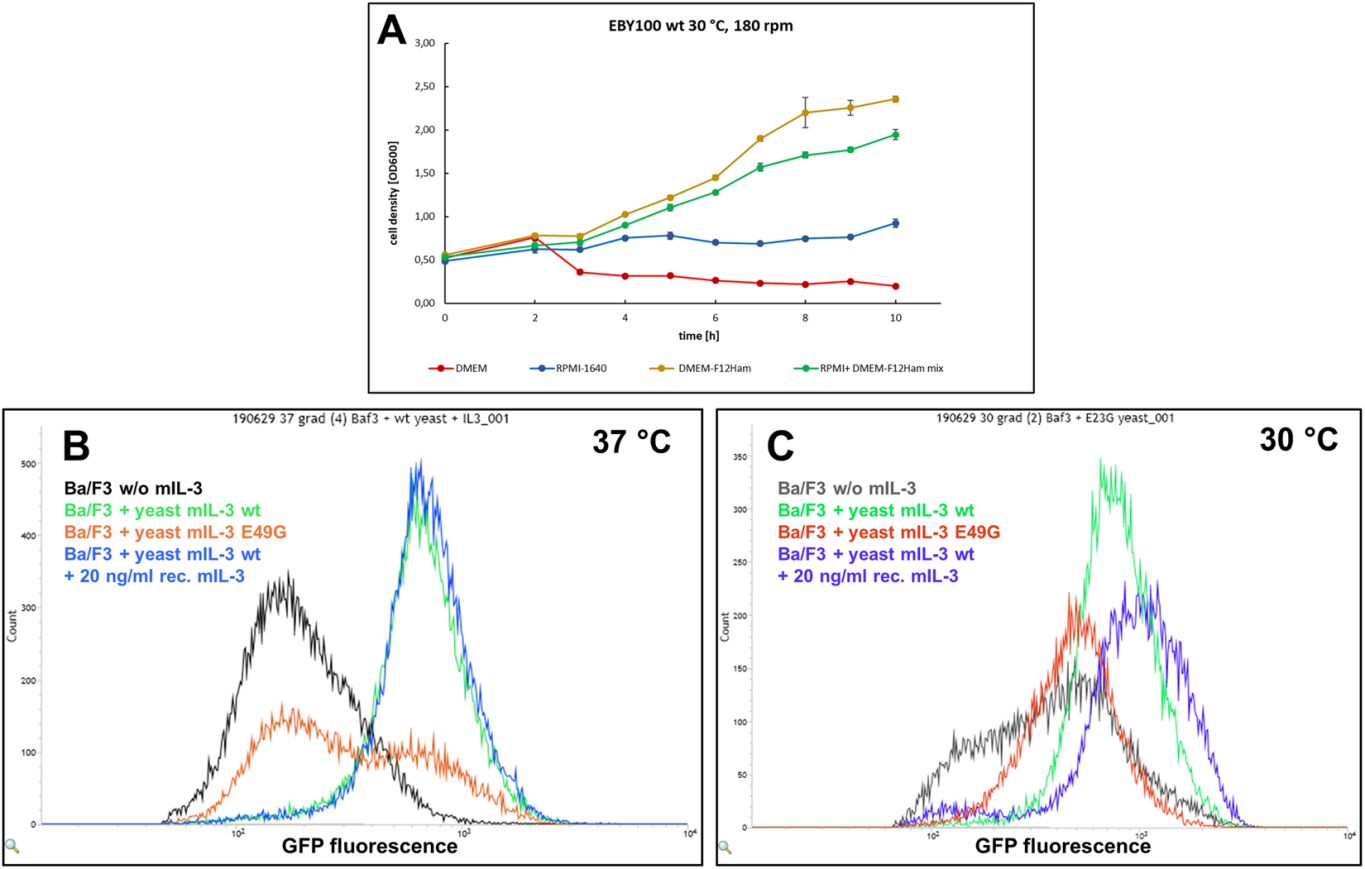
Investigation of optimal cocultivation conditions. (**A**) Growth curve of *S. cerevisiae* EBY100 in different mammalian culture media. Cell density was measured photometrically every hour for 10 h. (**B**) Cocultivation of Ba/F3-CIS-d2EGFP reporter cells with viable yeast cells in medium mix (50% RPMI-1640 + 50% DMEM-F12-Ham + 2% w/v galactose) at 37 °C (**B**) or 30 °C (**C**). Flow cytometry histograms depict the GFP fluorescence signal of the reporter cells only.

The challenging step in the process of establishing a reliable functional screen, which combines two living organisms from distinct kingdoms (Fungi and Animalia) is to identify culturing conditions suitable for both species (O_2_, CO_2_, humidity, medium, temperature, etc.). While *S. cerevisiae* prefers 30 °C and slightly acidic cell extract-based media^33^, mammalian cell lines need to be cultured by strict conditions – 37 °C, 5% CO_2_ in defined synthetic media with complex composition. In order to address this problem, we examined the yeast cell growth in three standard mammalian cell culture media – DMEM, RPMI-1640, and DMEM-F12 Ham. Sufficient cell growth was only observed in the DMEM-F12 medium (Fig. 4A), most likely due to the inorganic microelements, contained in the F12 nutrition mix. Since the Ba/F3 reporter cells are by standard cultured in RPMI-1640 medium, a 1:1 mixture of RPMI-1640 + DMEM-F12 Ham was tested, resulting in slightly reduced, but sufficient yeast cell growth and viability. Furthermore, the activation of the reporter cells by mIL-3 was not impaired in this medium mix, but stronger GFP fluorescence was measured (Fig. 2A, red curve). Reporter cell viability remained over 90% in the mixture of both culture media and mCherry fluorescence of the yeast cells upon galactose induction indicated successful expression and secretion (Supplementary Fig. S4).

### Cocultivation of mIL-3-secreting yeast cells and reporter cells in test tubes

Next, we tested the activation of reporter cells in the presence of live cytokine-secreting yeast cells at 37 °C (Fig. 4B) and 30 °C (Fig. 4C). Significant GFP signal separation between the Ba/F3 reporter cells, cocultured with the non-activating E49G mutant and the wt mIL-3 could be observed at 37 °C. The mIL-3 wt secretion level from the yeast cells was sufficient to induce maximal reporter cell activation, which could not be enhanced further by the addition of recombinant mIL-3 (Fig. 4B, blue histogram). In contrast, coculturing at 30 °C, resulted in high reporter cell mortality, accompanied by insufficient activation of the mammalian cells, even after stimulation with recombinant mIL-3 (Fig. 4C, blue histogram).

### Microfluidic co-encapsulation of yeast secretor cells and mammalian reporter cells in agarose-containing droplets

Utilizing a microfluidic system, we were able to co-encapsulate cytokine-secreting yeast cells together with mammalian reporter cells in monodispersed and homogenous agarose-containing droplets (Fig. 1). The droplet size is determined by the channel size of the microfluidic chip, as well as by the flow rates of the aqueous and oil phases. Using a commercially available flow-focusing microfluidic chip with 50 µm etch depth and flow rates of 4 µL/min and 35 µL/min for the aqueous and oil phases, respectively, droplets with 44 µm diameter on average and about 45 pL volume were generated (Supplementary Fig. S8A).

**Figure 5:**
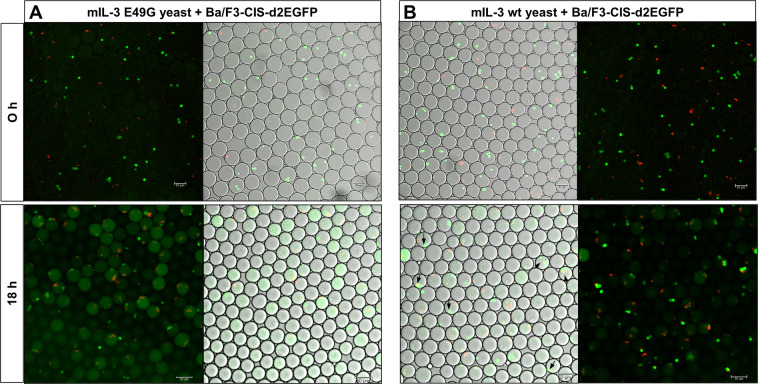
Microscopical analysis of co-encapsulated reporter and yeast cells. Ba/F3-CIS-d2EGFP were co-encapsulated with mIL-3 E49G (**A**) and mIL-3 wt (**B**) yeast cells in agarose-containing droplets and microscopically observed as w/o emulsion 0 h and 18 h after co-encapsulation. Black arrows indicate droplets were yeast and mammalian cells are localized in the same droplet.

Cell distribution in the droplets is dependent on the cell density of the encapsulated suspension and on the droplet size and can be predicted by Poisson’s equation^34^. Thus, optimal occupancy (one yeast and one mammalian cell) occurs only in a fraction of all droplets, affecting directly the FACS selection. In case low cell density of both cell types is used for co-encapsulation, the co-encapsulation efficiency drops drastically, resulting in a very high number of useless droplets. On the other side, high cell density leads to encapsulation of multiple yeast/mammalian cells in one droplet, and sorting of multiple yeast clones based on mammalian response induced by a single yeast cell (false positive). However, during subsequent co-encapsulation and selection cycles, the enriched false-positive yeast cells are eliminated to a large extend. Since our functional screen approach enables multiple screening rounds for specific enrichment of activating variants, more than one yeast cell per droplet is tolerated. Cell densities of 4 × 10^7^ yeast cells/mL and 2 × 10^7^ mammalian cells/mL were used for co-encapsulation, corresponding to an average of 0.9 yeast and 0.45 mammalian cells/droplet according to Poisson distribution. In order to determine the actual distribution of cells in the droplets, co-encapsulated cells in w/o emulsion were analyzed microscopically (Fig. 5, 0 h). While about 55% of all droplets were empty, approx. 23% of the occupied droplets contained one yeast and 50% one mammalian cell (Supplementary Fig. S8B). Generally, yeast cells are prone to flocculation^35^, which reflects the higher number of droplets with more than one yeast cell (38%) in comparison to the fraction of droplets with more than one mammalian cell (19%). Based on the microscopical analysis of 226 droplets, an average of 0.6 yeast and 0.41 mammalian cells per droplet were estimated, resulting in a theoretical co-encapsulation probability of about 15%. On account that only about 70% of the yeast cells exhibited sufficient mCherry fluorescence, the fraction of droplets containing an actively secreting yeast cell and a viable mammalian cell was determined by flow cytometry to be in the range between 8% and 10% of all droplets (Supplementary Fig. S5A). Furthermore, since nutrition and space are limited inside the picovolume droplets, co-encapsulation of a yeast cell with multiple mammalian cells could lead to false negative signals, due to reduced viability of the mammalian cells during the incubation step. To avoid losing desired variants due to false negative events, oversampling the yeast diversity during the co-encapsulation step at least 100 times is necessary. Nevertheless, with a generation speed of about 8 ***×*** 10^6^ droplets/h, at least 8 × 10^5^ yeast cells could be successfully co-encapsulated with a mammalian reporter cell in just one hour, making this system feasible for the screening of yeast-based libraries.

### Activation of reporter cells by co-encapsulation with mIL-3 secreting yeast cells in agarose droplets

**Figure 6:**
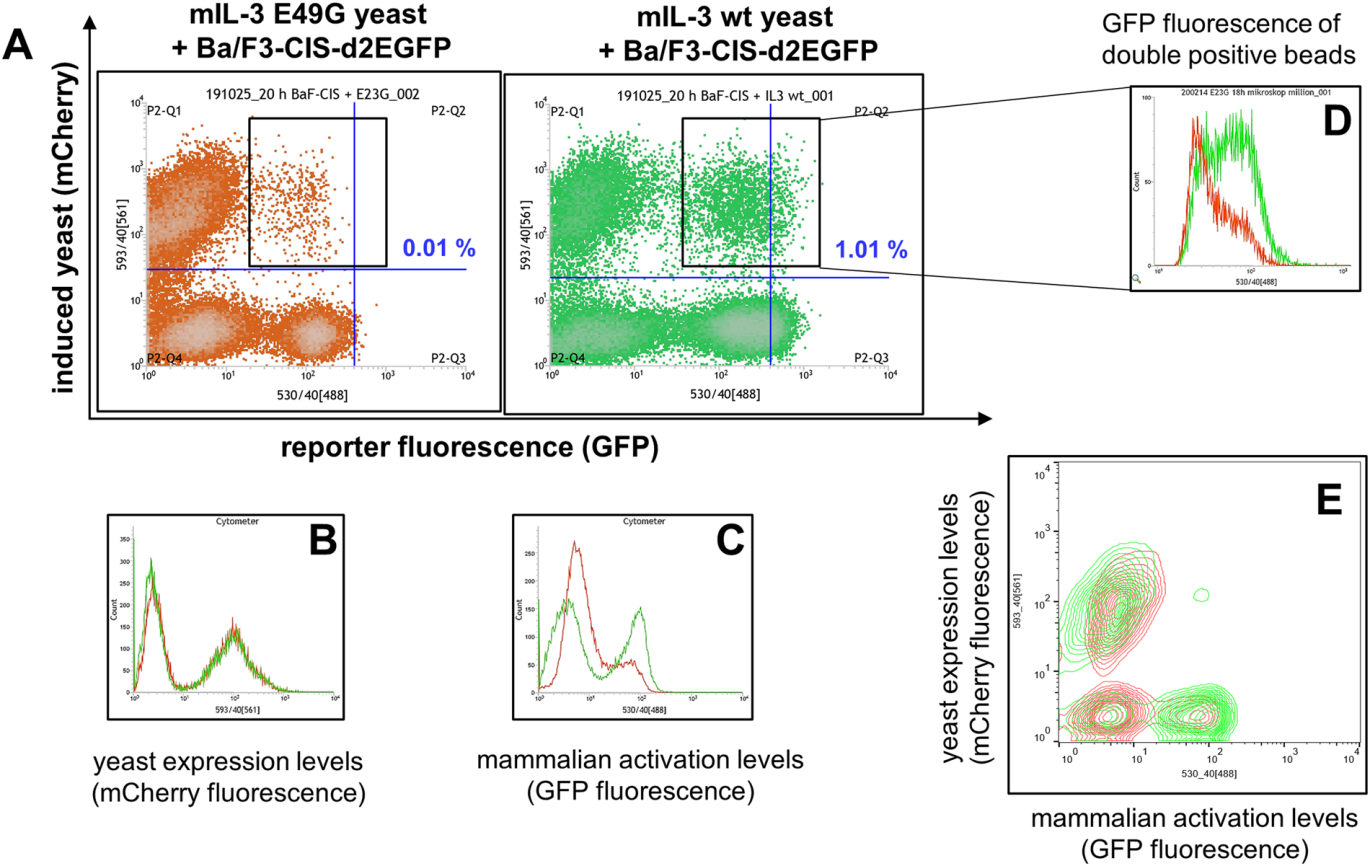
Flow cytometry of reporter cells co-encapsulated with mIL-3 E49G and mIL-3 wt yeast cells in agarose microbeads after 18 h of incubation at 37 °C. (**A**) Two dimensional FACS plot: x-axis represents GFP fluorescence of reporter cells (530/40[488] laser) and y-axis depicts the mCherry fluorescence of yeast cells (593/40 [561] laser). Gating strategy (blue) represents the double-fluorescent hydrogel microbeads identified as positive events during sorting (P2-Q2). (**B**) Overlay of the mCherry fluorescence signals of both samples. (**C**) Overlay of the GFP fluorescence signals of both samples. (**D**) Overlay of the GFP fluorescence intensity of the agarose microbeads containing mammalian and yeast cells (black box) from both samples. (**E**) Two-dimensional overlay of both samples (generated with FlowJo).

Murine Ba/F3 cells are generally mIL-3 dependent for cell growth. For this reason, the reporter cells were propagated with mIL-3-containing culture medium (10 ng/mL), leading to active GFP expression prior to co-encapsulation. This enabled the microscopically and flow cytometrical observation of the cell distribution directly after encapsulation as shown in Fig. 5 and Supplementary Fig. S5A. Directly after co-encapsulation with mIL-3 wt-, or mIL-3 E49G-secreting yeast cells, the mammalian cells in both samples showed strong GFP fluorescence, while no fluorescence background in the droplets could be observed (Fig. 5, 0 h). After incubation of the droplet-w/o-emulsion overnight at 37 °C, the droplets containing the non-functional mIL-3 variant exhibited diffuse fluorescence, spread throughout the whole droplet, implying cell lysis and release of the remaining GFP in the droplet (Fig. 5A, 18 h). In the case of the activating mIL-3 wt-secreting yeast, significantly more mammalian cells appear intact, leading to mainly cell-concentrated GFP fluorescence with higher intensity when yeast cells are colocalized in the droplet (Fig. 5B, 18 h). These observations were confirmed by flow cytometry analysis of the solidified agarose microbeads (Fig. 6). While the mCherry fluorescence intensity of both samples remains identical, indicating the same cytokine expression levels (Fig. 6B), the GFP fluorescence in case of co-encapsulation with wt mIL-3 is significantly stronger (Fig. 6C). By comparison of the GFP intensity of the double-fluorescent agarose microdroplets, the mIL-3 wt-containing microbeads exhibited noticeably stronger GFP fluorescence (Fig. 6D) with 1% lying in the high-stringency sorting gate (Fig. 6A P2-Q2). These results indicate sufficient viability and functionality of the mammalian reporter cells co-cultivated with the yeast cells under the tested conditions. Furthermore, a cell viability analysis of the encapsulated cells after co-cultivation of reporter and yeast cells in emulsion, confirmed no dramatic increase of mammalian cell mortality, with 60–70% viable reporter cells during FACS processing (Supplementary Fig. S5B).

### Enrichment of reporter-activating yeast after FACS of 1:10,000 mixture of mIL-3 wt and mIL-3 E49G

Gel microdroplet-fluorescence activated cell sorting (GMD-FACS) of *S. cerevisiae* was first described in 1995 as a tool to isolate slowly growing yeast cells from a mixed population^36^. Since particle size plays a decisive role in the sorting process, we first investigated the sorting efficiency using our agarose microbeads. To this end, GFP-producing EBY100 yeast cells were mixed with an excess of mCherry-producing EBY100 yeast cells and were encapsulated in agarose-containing microdroplets. GFP-positive agarose microbeads were sorted using a 200 µm nozzle (capable of sorting particles ≤ 50 µm). Flow cytometry after the expansion of the sorted yeasts revealed predominantly GFP-fluorescent yeast cells (Supplementary Fig. S1), confirming a highly efficient sorting process of the agarose microbeads, generated according to our encapsulation and sorting protocol.

**Figure 7:**
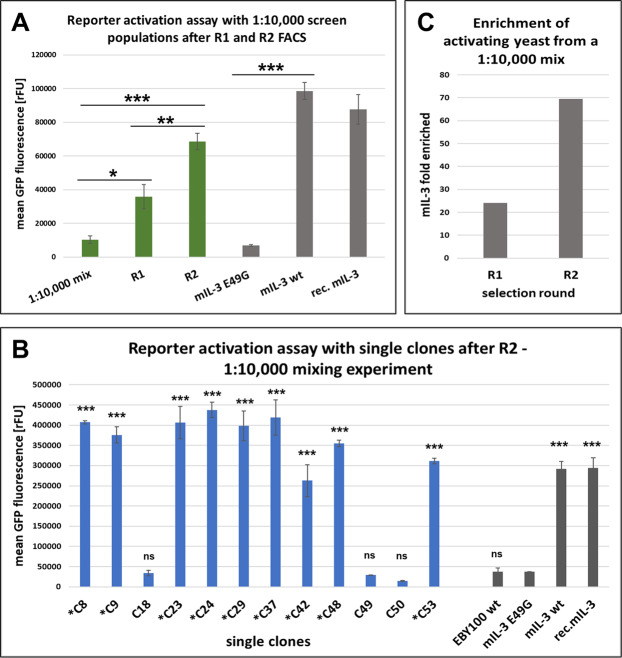
Analysis of selection outcome after functional selection of a 1:10,000 mixture of mIL-3 wt- and mIL-3 E49G-secreting yeast. (**A**) Mean GFP fluorescence signals of reporter cells after incubation with yeast culture supernatants of the induced population of the 1 to 10,000 mix of mIL-3 wt and mIL-3 E49G yeast cells and the populations after sorting round 1 and 2 (green). As controls (grey) reporter cells were cultivated with yeast supernatants of the non-functional E49G mutant, the biologically active mIL-3 wt, and 50 ng/mL recombinant mIL-3. All yeast cultures were inoculated at the same cell density and were cultured for 24 h at 30 °C. Final cell density was measured and the volume of yeast supernatant added to the mammalian cells was normalized to the final cell density. Statistical significance analysis was performed by One-way ANOVA and Turkey HSD post-hoc test (Supplementary Table S4). Relevant statistical significance between samples was marked on the graph. (**B**) Activation of reporter cells by incubation with culture supernatants of single yeast clones after the second round functional screening of the 1 to 10,000 mix. Single clones identified as mIL-3 wt by PCR are marked with an asterisk. All results represent the averaged mean fluorescence intensity from 3 biological replicates. Statistical analysis was performed by One-way ANOVA and Dunnett’s post-hoc multiple comparison test (Supplementary Table S5). Statistical significance between the reporter activation levels of the single clones and the negative control (mIL-3 E49G) was marked on the graph. (**C**) Fold enrichment of mIL-3 wt NGS reads in the populations of the first and second rounds of functional screening of the 1 to 10,000 mixture of mIL-3 wt- and mIL-3 E49G-secreting yeast.

In order to demonstrate the applicability of our system for a mammalian-based functional screen, we mixed the mIL-3 wt-secreting yeast cells with the yeast cells secreting the non-functional E49G mutant at a 1 to 10,000 ratio. Co-encapsulation with Ba/F3 reporter cells was performed as described above and only double-fluorescent agarose microbeads with strong GFP fluorescence were sorted (Supplementary Fig. S6). A reporter cell activation assay with culture supernatant of the bulk yeast populations revealed gradual enrichment of “activating yeast” (mIL-3 wt secretor) after the first and second sorting rounds (Fig. 7A), also confirmed by next-generation sequencing (Fig. 7C). PCR analysis of single clones after round 2 of encapsulation and FACS identified 9 of 54 clones as mIL3 wt (Supplementary Fig. S7) and supernatants from all of those clones induced reporter cell activation (Fig. 7B), confirming significant enrichment of wt mIL-3-secreting yeast from 0.01% to about 17%.

In order to determine the degree of enrichment reached by our functional selection approach, mixing experiments with 1:10, 1:100, and 1:1,000 ratios of both yeast types were additionally performed. Mixtures with 1:10 and 1:100 wildtype to mutant (E49G) mIL-3 demonstrated rapid enrichment of wt mIL-3 after a single functional selection cycle (Supplementary Fig. S10A). The 1:1,000 mixture was subjected to two consecutive selection rounds, resulting in gradual enrichment of the wildtype cytokine to about 26% of the population after R2 (Supplementary Fig. S10B,C). Enrichment degree in all experiments was limited to 15–26%. However, this is compatible with most screening campaigns since sufficient number of functionally active single clones could easily be identified by single clone analysis. If necessary, further increase in the enrichment degree could be achieved by decreasing the yeast cell density used for the co-encapsulation process. This would minimize encapsulation of multiple yeast cells in the same droplet and thus, sorting of false positive yeast cells would be avoided.

## Discussion

With the rapid expansion of the biopharmaceutical market, the demand for straightforward, cost-effective and time-saving discovery technologies for the generation of potent functional biologics has increased tremendously. The interest in the establishment of novel high-throughput functional screening methods, which surpass the limitations of the current state of the art technologies has raised, leading to the development of commercial platforms like Cyto-Mine from SphereFluidics^37^, Beacon Optofluidic Platform from Berkeley Light, and xPloration Screening Platform from xCella Biosciences^38^. With the advantages of sample miniaturization and encapsulation in distinct microdroplets, microfluidic-assisted screening has emerged as a promising next-generation approach.

Here we demonstrate a novel strategy for mammalian reporter cell-based functional screening by co-encapsulation of viable mammalian reporter cells with yeast secretor cells in millions of agarose-containing microdroplets. Our system is compatible with ultra-high-throughput screening utilizing a commercially available FACS device without need of extensive optimization and adaptation. With the help of a proof-of-principle activator-reporter system, we demonstrate the feasibility of the system for functional screening of protein variants produced by yeast. *S. cerevisiae* as a library host combines the advantages of a microbial expression system, which is characterized by high transformation efficiencies and fast growth with those of eukaryotes since they possess an unfolded protein response machinery for elimination of misfolded proteins prior to secretion^39^. In addition, it is compatible with both immune and combinatorial libraries^40,41^. Simultaneous secretion and display of the library variants on the yeast surface could be achieved by utilizing an inefficient ribosomal skipping (F2A)^42^, enabling alternating affinity-based^43^ and functional selection using the same mammalian target (reporter) cell line. Furthermore, human-like N-glycosylation could successfully be engineered in *S. cerevisiae*^44^, which is an important factor influencing the bioactivity, physicochemical properties, and pharmacokinetics of the majority of biologics^45–47^. Lastly, *S. cerevisiae* is a robust organism, capable of fast expansion after encapsulation and sorting of agarose microbeads, which enables functional screening in multiple rounds or different setups without risk of losing desired candidates due to low cell viability.

In comparison, several similar screening approaches have been published in recent years, which once again emphasizes the high demand in this field. In 2017, Fang *et al*. proposed an affinity-based antibody screening platform, based on co-encapsulation of antibody-secreting *Pichia pastoris* and fixed mammalian target cells in agarose microdroplets^48^. This study demonstrated that GMD-FACS is a reliable selection method and classical antibody staining procedures can be applied for the specific fluorescent detection of target-bound antibodies in the agarose microbeads. However, since this procedure was not optimized for viable mammalian cells, functional antibody screening would not be feasible. Nevertheless, we acknowledge the potential of utilizing *P. pastoris* for efficient secretion of full-length IgG antibodies^49^ and we are confident that our functional screening system could easily be adapted for co-encapsulation with antibody-secreting *P. pastoris*.

Another system for microfluidic-based functional antibody selection was introduced by Zheng *et al*. in 2018. In this study, the authors co-encapsulated phage-secreting *E. coli* and mammalian reporter cells in medium-containing aqueous droplets and were able to detect reporter cell activation by agonist antibody fragment (scFv) presented on the phage surface^50^. This work expands the compatibility of droplet-based functional screening towards one of the best-established screening approaches, phage display^51^. We are convinced that in combination with a straightforward ultra-high-throughput GMD-FACS (by addition of agarose to the encapsulation medium) this approach would be feasible for phage display-based selection of agonistic antibody variants, bypassing the need of double emulsion generation, addressed by the authors.

A further example, demonstrating the versatility of droplet-based functional screening utilizing mammalian reporter cells is the study of Yaginuma *et al*. (2019). In a similar to our system fashion, they co-encapsulated peptide-secreting *S. cerevisiae* with mammalian reporter cells, responding to activation of the G-protein coupled receptor hGLP1R^52^. The fluorescence read-out, in this case, was achieved by expression and secretion of β-galactosidase (LacZ) as a reporter enzyme, which converts a non-fluorogenic substrate, added to the droplets, into a fluorescent molecule. Selection of agonistic peptide variants from a randomized peptide library was performed by co-encapsulation of the peptide-secreting yeast with the reporter cells and manual microscopical observation of the droplets, followed by isolation of the highly fluorescent droplets by a micromanipulator. This study illustrates the applicability of droplet-based cocultivation screens utilizing viable mammalian cells for addressing challenging targets as GPCRs, where the generation of reliable soluble protein for standard affinity-based screens is not trivial. However, the throughput of this system is mainly limited by the microscope-assisted isolation of fluorescent droplets.

In summary, a murine Ba/F3 reporter cell line, expressing green fluorescent protein (GFP) upon stimulation with murine interleukin-3 (mIL-3) was constructed for a proof-of-concept study. Co-encapsulation of wt mIL-3 secreting yeast cells with Ba/F3 reporter cells in agarose-containing droplets resulted in strong activation of the reporter cells, whereas after co-encapsulation with a non-functional mutant only moderate GFP signal was detectable. Co-encapsulating a 1 to 10,000 mixture of wt mIL-3 and the mutant secreting yeast cells with the mammalian reporter cell line and sorting for double-fluorescent events resulted in rapid enrichment of yeast cells secreting biologically active wt mIL-3 molecules. In conclusion, we believe that our novel platform for an ultra-high-throughput functional screening could enable and accelerate the discovery of new functional biologics, including cytokines, agonistic or internalizing antibodies, and soluble receptors^53^.

## Materials and methods

### Generation of the mammalian reporter cell line - Ba/F3-CIS-d2EGFP

The coding sequence for d2EGFP (destabilized enhanced Green Fluorescent Protein) was amplified by polymerase chain reaction from plasmid pd2EGFP-N1 (Takara Bio, formerly Clontech, Mountain View, CA, USA) using primers JB46F1 (5′-CGAAGCTTGGCATTCCGGTACTGTTGGTAAAGCCACCATGGTGAGCA-3′) and JB47R1 (5′- ACGGATCCCTTAAGATACATTGATGAG-3′). PCR amplicon was digested with *Hind*III and *Bam*HI and cloned into plasmid pGL4.17 (Promega, Madison, WI, USA) after removal of the luciferase gene by excision with *Hind*III and *Bam*HI.

The resulting construct pGL4.17-d2EGFP was subsequently subjected to a partial digest with *Kpn*I and *Hind*III to allow for the cloning of a fragment containing the regulatory promoter region from the STAT5-responsive human CIS gene. The CIS promoter fragment was excised from reporter gene plasmid pGV-CIS-luc^54^, kindly provided by Dr. A. Yoshimura, Kyushu University, Fukuoka, Japan) using *Kpn*I and *Hind*III and placed upstream of the d2EGFP coding sequence to yield pGL4.17-CIS-d2EGFP.

Plasmid pGL4.17-CIS-d2EGFP was introduced into the interleukin-3-dependent murine pro-B cell line Ba/F3 by electroporation as described previously^55^. Neomycin-resistant cell clones were selected by cultivation in the presence of 1 mg/mL G418 for two weeks. Obtained G418-resistant cell clones were tested for mIL-3-inducible d2EGFP expression by 3 h cytokine starvation of 10^5^ cells in 100 µL aliquots followed by stimulation for 12 h with 10 ng/mL recombinant murine interleukin-3 (rec. mIL-3) (Invigate GmbH, Jena, Germany). Inducible EGFP expression was analyzed by flow cytometry employing a CyFlow Space device (Sysmex Partec GmbH, Görlitz, Germany) and the software Cyflogic 1.2.1 (http://www.cyflogic.com), resulting in the identification of the mIL-3-responsive reporter cell line Ba/F3-CIS-d2EGFP.

Reporter cells were further cultured in RPMI-1640 medium supplemented with 10% FBS and 1% Penicillin-Streptomycin solution (Pen/Strep) (Sigma Aldrich P4333), 10 ng/mL recombinant mIL-3 were added to the medium for normal culturing and expansion of the cells.

Responsiveness of the Ba/F3-CIS-d2EGFP reporter cells to mIL-3 was examined using a dilution row of recombinant mIL-3. Therefore, 10^5^ reporter cells/well were washed twice with PBS, resuspended in 100 µL/sample culture medium with 10% FBS and 1% Pen/Strep and plated in a 96-well plate. Recombinant mIL-3 was serially diluted (1:2) in the same culture medium (0.005–82 ng/mL) in a volume of 100 µL/sample. The mIL-3-containing medium was added to the cell suspension in triplicates and the cells were incubated in a humidified atmosphere at 37 °C, 5% CO_2_ overnight (18–20 h). Mean GFP fluorescence of the population was measured on BD Accuri C6 flow cytometer and cell populations of some concentrations were additionally analyzed on BD Influx Cell Sorter.

### Yeast strains, media and reagents

*Saccharomyces cerevisiae* strain EBY100 [MATa URA3-52 trp1 leu2Δ1 his3Δ200 pep4:HIS3 prb1Δ1.6 R can1 GAL (pIU211:URA3)] (Thermo Fisher Scientific) was utilized for the secretion of murine IL-3. SD (synthetic dropout) medium was composed of 8.6 g/L NaH_2_PO_4_ × H_2_O, 5.4 g/L Na_2_HPO_4_, 1.7 g/L yeast nitrogen base without amino acids, 5 g/L ammonium sulphate, 1.3 g/L dropout amino acids mix (w/o leucine) and 20 g/L dextrose. SG medium was prepared identically except for the substitution of dextrose sugar with galactose. Phosphate-buffered saline (PBS) contained 8.1 g/L NaCl, 1.13 g/L Na_2_HPO_4_, 0.75 g/L KCl, and 0.27 g/L KH_2_PO_4_.

### Generation of mIL-3-secreting yeast

For the generation of *S. cerevisiae*-secretion vector a pYD1 plasmid (Yeast Display Vector Kit, version D, #V835-01, Thermo Fisher Scientific), comprising GAL1-promoter, Leu2 (Beta-isopropyl malate dehydrogenase), and ampicillin resistance was used. mIL-3 gene was amplified by PCR and complementary overhangs (30–40 bp) were introduced for gap repair cloning procedure. Gene sequence of mCherry fluorescent protein with T2A ribosomal skipping peptide on the 5´-end was generated by PCR and complementary overhangs were introduced. Secretion vector comprising constructs in the following order: GAL1-promoter, app8-secretion signal, mIL-3 wt gene, T2A ribosomal skipping peptide and mCherry fluorescent protein (Fig. 3A) was generated in single-step gap-repair cloning in *S. cerevisiae* EBY100.

A non-functional mIL-3 variant (E49G) was generated identically using corresponding mismatch primers for the PCR on the wt mIL-3 gene.

For gap repair cloning EBY100 yeast cells were prepared according to Benatuil and colleagues^56^. pYD-Leu-TRAP vector (Supplementary Fig. S9) was digested with *Nde*I and *Pme*I and the linearized backbone was extracted from an agarose gel using Wizard SV Gel and PCR Clean-Up System (Promega). For gap repair cloning,1 µg purified backbone was mixed with 300 ng mIL-3 insert and 500 ng T2A-mCherry insert. 100 µL electrocompetent EBY100 cells were added to the DNA and incubated for 10 min on ice. Cells were electroporated using BioRad electroporation system, according to the protocol of Bernatuil *et al*. and incubated for 1–2 h in 2–3 mL YPD medium (20 g/L dextrose, 20 g/L tryptone and 10 g/L yeast extract) at 30 °C and 180 rpm. Cells were pelleted by centrifugation at 4000 rpm for 3 min and washed once with sterile PBS. Finally, the cells were plated on selective minimal medium agar plates and incubated for 2–3 days in a static incubator at 30 °C.

### Verification of mIL-3 secretion and activation of reporter cells

For the verification of successful secretion of active (wt) and inactive (E49G) mIL-3, the corresponding yeast clones were inoculated in an autoinduction medium (25% SD + 75% SG) and cultured at 30 °C, 180 rpm for 48 h. Yeast supernatant was harvested by centrifugation for 10–15 min at 17 000 × g and sterile filtered using a 0.2 µm sterile filter. The pH of the supernatant was measured and neutralized using 1 M HEPES buffer. 1 × 10^5^ reporter cells/sample (Ba/F3-CIS-d2EGFP) were harvested and washed 2 times with 5 mL sterile PBS to remove bound recombinant mIL-3. The cells were resuspended in 100 µL/sample RPMI-1640 + 20% FBS and 1% Pen/Strep solution and plated in a 96 well plate. 100 µL yeast supernatant were added to each well and the cells were incubated at 37 °C in a humidified atmosphere with 5% CO_2_ for 18–20 h. GFP fluorescence intensity was measured on the next day using flow cytometry.

### Growth and induction of yeast cells in mammalian medium

Yeast cells (*S. cerevisiae* EBY100 or EBY100-mIL-3 wt) were inoculated at a cell density of 5 × 10^6^ cells/mL (OD_600_ = 0.5) in 3 mL mammalian medium (DMEM high glucose, RPMI-1640, DMEM-F12 Ham, or mix of 50% RPMI-1640 + 50% DMEM-F12 Ham) and cultivated at 30 °C or 37 °C, 180 rpm. OD_600_ was measured every 60 min.

Yeast induction (EBY100-mIL-3 wt) was determined by measuring the fluorescence intensity of the intracellular expression marker (mCherry) by flow cytometry after 24 h of cultivation in the corresponding mammalian medium, supplemented with 2% galactose. The percentage of mCherry-fluorescent cells was used as a measure of yeast induction.

### Cocultivation of mammalian reporter cells and mIL-3-secreting yeast in medium

Yeast cells, secreting wt mIL-3 or E49G mIL-3 were expanded and induced overnight in autoinduction medium (25% SD + 75% SG). 1 × 10^7^ yeast cells were mixed with 1 × 10^6^ Ba/F3-CIS-d2EGFP reporter cells in 1 mL mammalian medium mix (50% RPMI-1640 + 50% DMEM-F12 Ham), supplemented with 10% FBS, 2% galactose and 1% Pen/Strep and transferred in a 50 mL centrifugation tube. The vessels were sealed with a gas-permeable membrane (VWR 60941-084) in order to prevent evaporation of the medium. Cells were cocultured overnight at 37 °C, 110 rpm in a humidified atmosphere with 8% CO_2_ or at 30 °C, 180 rpm. Activation of the reporter cells was estimated based on the GFP fluorescence measured on BD Influx cell sorter.

### Single-cell co-encapsulation using droplet microfluidics

For the encapsulation in hydrogel-microdroplets, fresh encapsulation medium was prepared each time: 3% w/v ultra-low gelling temperature agarose (Sigma Aldrich A5030) was suspended in 2 mL mammalian medium mix (50% RPMI-1640 + 50% DMEM-F12 Ham) and the suspension was heated up in a microwave until the agarose completely dissolved. The agarose solution (3% w/v) was then tempered in a water bath at 37 °C. All other components of the encapsulation medium were tempered at 37 °C, as well, to prevent solidification of the agarose. Encapsulation medium consisted of 0.75% agarose, 20% FBS, 20% Opti-Prep density gradient medium (Sigma Aldrich D1556), 2% galactose and 1% Pen/Strep in medium mix (50% RPMI-1640 + 50% DMEM-F12 Ham) (Supplementary Table S3).

Cytokine-secreting yeast cells (wt mIL-3 or E49G mIL-3) were propagated and simultaneous expression induction was achieved utilizing selective autoinduction medium (25% SD + 75% SG) at 30 °C, 180 rpm. Cell density was estimated using a photometer (OD_600_ = 1 corresponds to 1 × 10^7^ yeast cells/mL) and 4 *×* 10^7^ yeast cells were pelleted and washed once with sterile PBS. Cells were resuspended in 1 mL of encapsulation medium and kept at 37 °C during the whole encapsulation process to prevent agarose solidification.

Ba/F3-CIS-d2EGFP reporter cells were cultivated as described above. Cell density and viability was estimated using TC20™ Automated Cell Counter (Bio-Rad) and Trypan Blue stain. 2 × 10^7^ viable cells were harvested by centrifugation at 800 rpm for 5 min at room temperature and washed twice with sterile PBS. Cells were resuspended in 1 mL encapsulation medium and kept at 37 °C as well.

Co-encapsulation was performed with a µEncapsulator system (Dolomite Bio) using a 50 µm fluorophilic 2 Reagent Droplet Chip (Dolomite Bio 3200445). 3 M™Novec™7000 engineered fluid, supplemented with 2% Pico-Surf surfactant (Sphere Fluidics) was used as a continuous phase. Prior to cell encapsulation, the microfluidic system was equilibrated to 37.5 °C using the integrated TCU device to prevent clogging of the chip by solidified agarose. 100 µL of the yeast cells, resuspended in agarose-containing encapsulation medium were applied on the µEncapsulator Sample Reservoir Chip (Dolomite Bio 3200444) and 100 µL of the reporter cells were applied on the second aqueous channel. Co-encapsulation was performed at flow rates of 3–4 µL/min for both cell suspensions and 30–40 µL/min for the continuous phase, thus resulting in encapsulation rates of 1.4 × 10^5^ yeast cells and 7 × 10^4^ reporter cells per minute. As a running fluid for the pressure pumps of the aqueous channels, plain 3 M Novec 7000 engineered fluid (without surfactant) was used. The generated droplets were collected in sterile 1.5 mL tubes (also tempered at 37 °C all the time).

After the cell samples (100 µL each cell type) were completely encapsulated and no droplet formation could be observed, the microfluidic system was paused and the emulsion was transferred to a rotating incubator and incubated for 17–18 h at 37 °C, 110 rpm, in a humidified atmosphere and 8% CO_2_.

### Mixing Experiments using wt mIL-3- and E49G mIL-3-secreting yeast

To demonstrate that our platform is suitable for selection based on the functionality of the secreted molecules, mixing experiments with the biologically active and inactive mIL-3-variants were performed. To this end, yeast cells secreting the active wt mIL-3 were mixed to a 1:10,000 ratio with the yeast which secretes nonfunctional mIL-3 E49G mutant. Yeast cells were expanded and pre-induced separately and after cell density determination mixed in the corresponding ratio. Co-encapsulation with the reporter cells was performed as described above.

For the 1:10,000 proof-of-concept approach, 3.2 × 10^7^ yeast cells were co-encapsulated with 1.6 × 10^7^ reporter cells and incubated overnight.

### Agarose bead isolation and FACS sorting

Prior to analysis and sorting, the emulsion with the co-encapsulated cells was cooled for 15–20 min on ice in order to allow the agarose to solidify. The clear oil phase under the emulsion was removed and the emulsion was broken using 150 µL PFO (1H,1H,2H,2H-perfluoro-1-octanol Sigma Aldrich). After short centrifugation (100 rpm, 10 s), the agarose microbeads were visible as a clear, yellow upper phase. The lower phase, containing the PFO and the residual fluorocarbon oil, was removed and the agarose beads were resuspended carefully in 1 mL sterile, cold PBS. Prior to FACS, the resuspended agarose beads were filtered using a 70 µm cell strainer to prevent clogging of the sample line or nozzle.

Sorting of agarose beads was performed on BD Influx cell sorter, equipped with a 200-micron nozzle tip (BD Biosciences), with a 6 kHz drop frequency. BD FACSFlow sheath fluid (Fisher Scientific) was used with 3.0 psi sheath pressure.

After each sorting round, the sorted yeast-containing agarose beads were plated on selective SD agar plates and incubated for 3 days at 30 °C in a static incubator. The colonies were harvested from the plates using sterile SD and stored at 4 °C until further usage.

### Enrichment analysis using bulk populations after each sorting round

Monitoring of sorting efficiency was performed using bulk populations after each sorting round. To this end, yeast cells of the non-sorted mix and of the corresponding sorting rounds were inoculated at a cell density of 1 × 10^7^ cells/mL in test tubes containing 2 mL of DMEM-F12 Ham medium, supplemented with 2% galactose and 1% Pen/Strep. Cells were cultivated for 24–48 h at 30 °C, 180 rpm and after cell density measurement, the yeast cultures were centrifuged for 15 min, 17 000 × rcf in 1.5 mL Eppendorf tubes. The supernatant (1 mL) was transferred to a new tube and pH was neutralized with 100 µL 1 M HEPES buffer. 1 × 10^5^ reporter cells/sample were harvested and washed twice with sterile PBS. Reporter cells were resuspended in 100 µL RPMI medium, supplemented with 20% FBS and 1% Pen/Strep. The volume of yeast supernatant (max. 100 µL), added to the reporter cells, was normalized to the final cell density of the yeast culture. Reporter cells were subsequently incubated overnight (15–18 h) at 37 °C, 5% CO_2_ in a 96 well plate. Enrichment of functional wt mIL-3-secreting yeast cells was verified by the measurement of elevated GFP-fluorescence signal, normalized to background fluorescence of non-activated Ba/F3 reporter cells. GFP fluorescence signal (10,000 viable cells/sample) was determined using BD Accuri C6 flow cytometer. Statistical analysis was performed by One-way ANOVA followed by Honestly Significant Difference test (Tukey HSD post-hoc) with the help of GraphPad Prism software. GFP signals of all samples were statistically compared and the statistical significance (P value) was determined (Supplementary Table S4).

### PCR screen of single clones from mixing experiments

For facile analysis of the sorting process in case of mixed mIL3 wt and E49G, a mIL-3 wt- specific primer was designed (Table S2). Colony PCR with single clones after the second sorting round was performed, where a 400 bp PCR product indicated the presence of wt mIL-3 coding DNA sequence. For the colony PCR, each colony was resuspended in 50 µL 20 mM NaOH and was heated at 98 °C for 15 min in 200 µL PCR tubes. After cooling down, the samples were centrifuged to pellet the cell debris and 1 µL supernatant per clone was used as a template. PCR was performed utilizing Taq DNA Polymerase (Qiagen) according to the standard manufacturer’s protocol. PCR consisted of initial denaturation step (94 °C - 3 min); 30 cycles of denaturation (94 °C – 30 s), annealing (60 °C – 30 s), and elongation (72 °C – 30 s); final elongation (72 °C – 5 min). Colony PCR was analyzed using agarose gel electrophoresis.

Positive single yeast clones were further analyzed by means of reporter cell activation assay using supernatants of each single clone as described above. Mean GFP fluorescence was determined using BD Accuri C6 flow cytometer and was normalized to background fluorescence of non-activated Ba/F3 reporter cells. Statistical analysis was performed by One-way ANOVA followed by Dunnett’s test for multiple comparison of the single clones with the negative control (mIL-3 E49G). The statistical significance (P value) was estimated using the indicated methods in GraphPad Prism software (Supplementary Table S5).

### Next-Generation Sequencing (NGS)

The efficiency of our functional screening approach was analyzed using NGS of the mixed 1:10,000 mIL-3 wt and mIL3-E49 yeast population, as well as of the bulk populations after each selection round. To this end, yeast cell populations were propagated for 24 h at 37 °C in SD medium. 1 × 10^8^ cells were centrifuged and plasmid preparation was performed using Zymoprep Yeast Plasmid Miniprep Kit (Zymo Research) according to the manufacturer’s protocol. Specific oligonucleotides, comprising partial Illumina adapters and a DNA barcode were designed (Supplementary Table S2). PCR was performed using 0.5 µL plasmid preparation from each population as a template utilizing Q5 Polymerase (NEB) according to the standard protocol (NEB). PCR amplicons were purified using Wizard SV Gel and PCR Clean-Up System (Promega) following the manufacturer’s protocol. Samples were sent for NGS to GENEWIZ (Leipzig, Germany) and sequencing data analysis was performed using Geneiuos 2020.1.

## Supplementary information

Supplementary information.
