# Supplementary material for: FACS-Based Functional Protein Screening via Microfluidic Co-encapsulation of Yeast Secretor and Mammalian Reporter Cells
Source: Sci Rep. 2020 Jun 23;10:10182. doi: 10.1038/s41598-020-66927-5 (PMC7311539; doi:10.1038/s41598-020-66927-5)
Supplement: Supplementary file 1 — Supplementary information. [file 41598_2020_66927_MOESM1_ESM.pdf]

Supplemental

FACS-Based Functional Protein Screening via Microfluidic Co-encapsulation of Yeast Secretor and Mammalian Reporter Cells

Desislava Yanakieva[1], Adrian Elter[1,4], Jens Bratsch[2], Karlheinz Friedrich[2], Stefan Becker[3], Harald Kolmar[1,4*]

[1]Institute for Organic Chemistry and Biochemistry, Technical University of Darmstadt, Alarich-Weiss-Strasse 4, D-64287 Darmstadt, Germany

[2]Institute for Biochemistry II, University Hospital Jena, 07743 Jena, Germany

[3]Protein Engineering and Antibody Technologies, Merck Healthcare KGaA, Frankfurter Straße 250, D-64293 Darmstadt, Germany

[4]Merck Lab @ Technische Universität Darmstadt, Alarich-Weiss-Strasse 8, D-64287 Darmstadt, Germany

[*]To whom correspondence should be addressed:

Harald Kolmar: Institute for Organic Chemistry and Biochemistry, Technische Universität Darmstadt, Alarich-Weiss-Straße 4, D-64287 Darmstadt, Germany

Email: Harald.Kolmar@TU-Darmstadt.de

Keywords: functional screen, droplet-based microfluidics, hydrogel, agarose microbeads, yeast library, mammalian reporter cell, cytokines, murine IL-3, FACS

Supplementary Figures

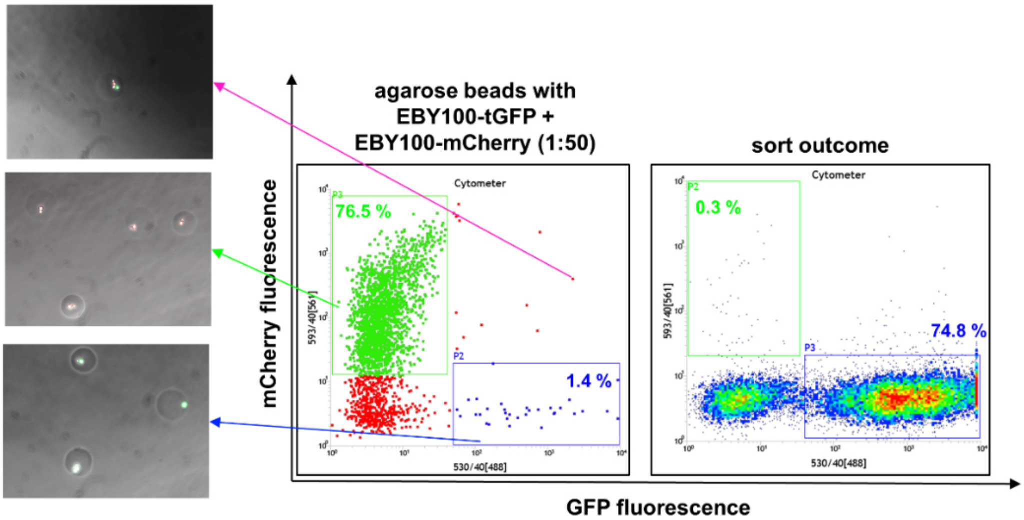

Figure S1. Sorting of agarose microbeads by FACS. Green fluorescent yeast cells (EBY100-tGFP) were mixed with red fluorescent yeast cells (EBY100-mCherry) at a 1:50 ratio. Co-encapsulation in agarose containing droplets was performed using 7.5 × 10^6 yeast cells per mL encapsulation medium in order to minimize co-encapsulation of two different yeast types. Agarose microbeads with encapsulated yeast cells were sorted by FACS. Sorting efficiency was analyzed by sorting mCherry-positive (green), GFP-positive (blue), and double-positive (magenta) events, followed by microscopical observation. Agarose microbeads in the blue gate (GFP-positive) were sorted and plated on agar plates. Analysis of sort outcome was performed after propagation and of the sorted yeast (plot on the right).

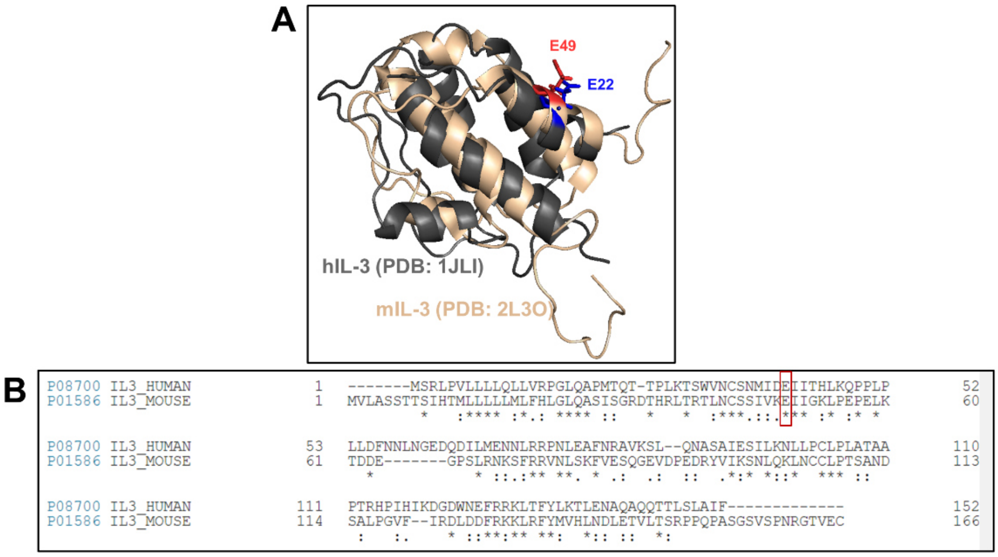

Figure S2. Structural and sequential comparison of human and murine IL-3. (A) Protein structures were obtained from PDB and aligned using PyMol. **(B)** Amino acid sequences were obtained and aligned using UniProt data base.

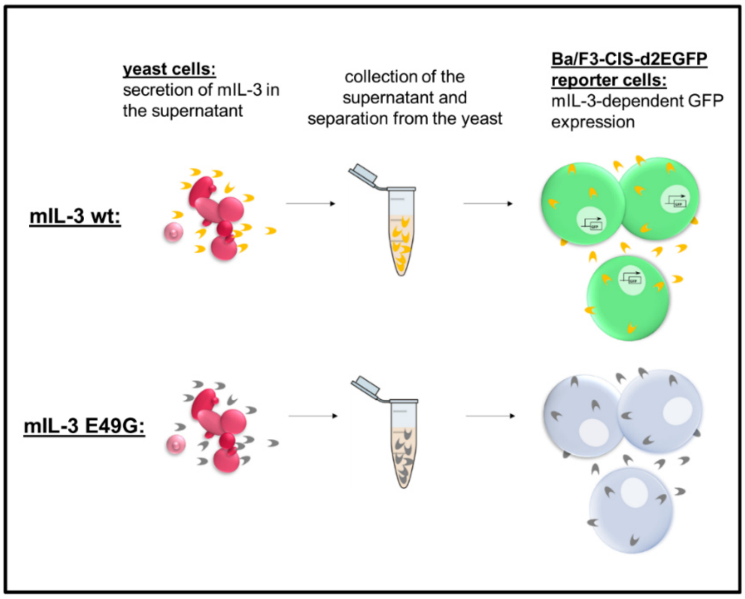

Figure S3. Reporter cell activation assay using yeast supernatant. Cytokine-secreting yeast cells (mIL-3 wt or mIL-3 E49G) are cultured until sufficient cell density and mCherry-fluorescence (expression control) are reached. Cytokine-containing yeast supernatant is harvest by centrifugation and added to the reporter cells. GFP fluorescence is measured by flow cytometry after 16-18 h incubation at 37 °C, 5 % CO_2.

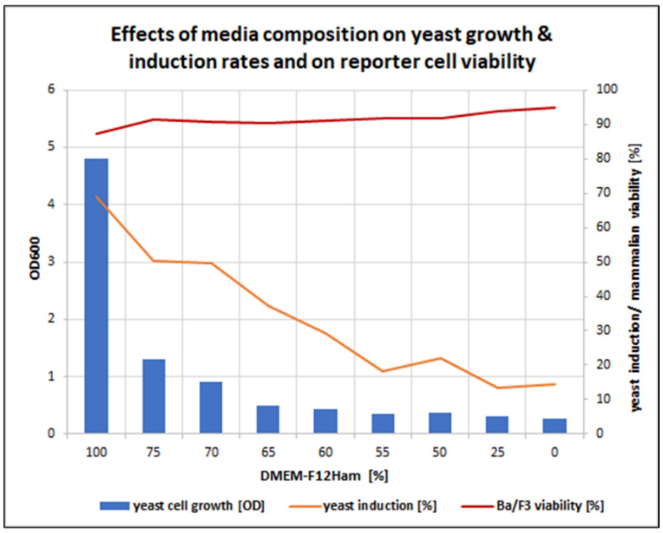

Figure S4. Medium-dependent yeast cell growth and induction rates, as well as mammalian reporter cell viability. DMEM-F12 Ham medium was mixed with RPMI-1640 in different ratios (0 – 100 %) and supplemented with 2 % galactose and 1 % Pen/Strep. Yeast cell density (OD_{600}) was determined after 24 h of incubation at 30 °C, 180 rpm and induction levels were determined as percent mCherry positive yeast cells by flow cytometry. Reporter cell viability was measured after staining with propidium iodide (PI) by flow cytometry and percent viable cells was plotted on the graph for each medium composition.

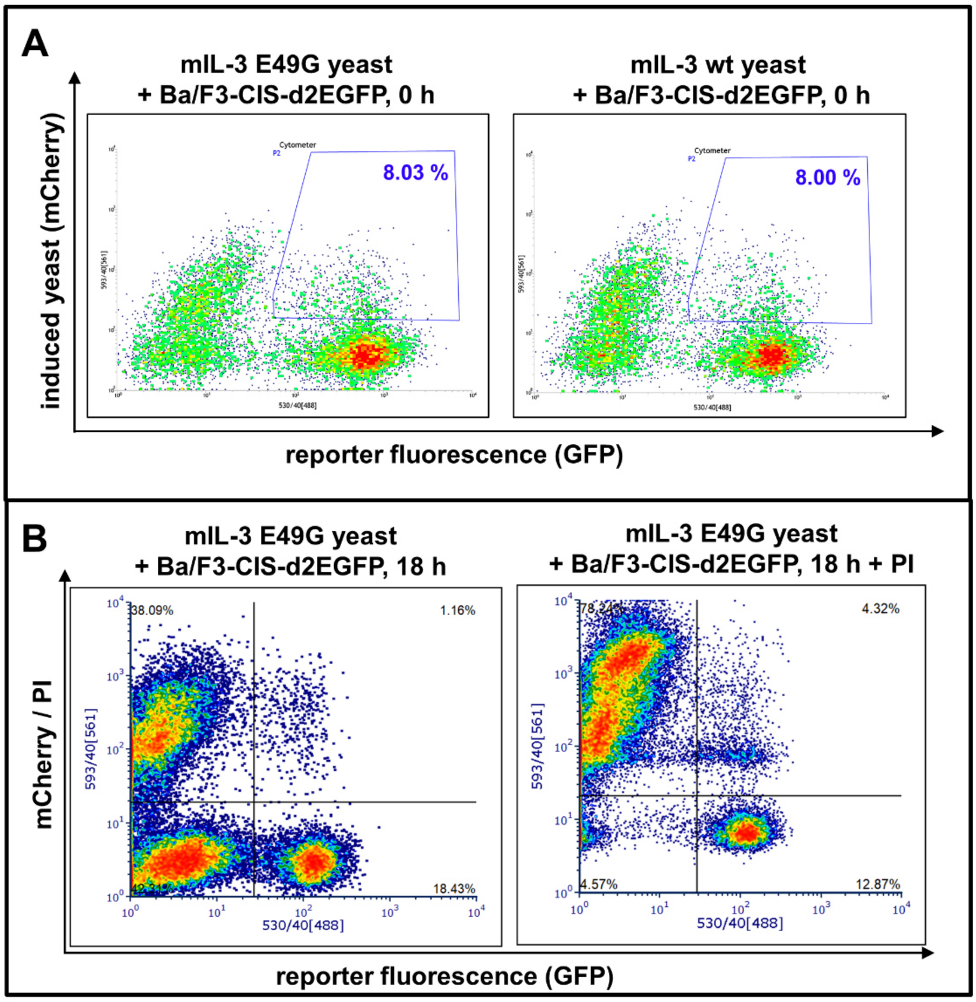

Figure S5. (A) Analysis of co-encapsulation efficiency directly after co-encapsulation. Agarose containing droplets were generated by co-encapsulation of mammalian reporter cells with either mIL-3 wt- or mIL-3 E49G-secreting yeast cells, which were priorly induced overnight in autoinduction medium. Directly after encapsulation the w/o emulsion was cooled for 15 min on ice for agarose microbeads formation. Agarose microbeads were subsequently analyzed on BD Influx ™ FACS. Population in the blue gate represents double positive agarose microbeads, containing at least one mCherry-positive yeast and one GFP-positive reporter cell. **(B) Analysis of reporter cell viability after co-encapsulation with yeast cells and 18 h of incubation in w/o emulsion.** Following agarose solidification, agarose microbeads were recovered in PBS buffer. Dead cells were stained with propidium iodide and agarose microbeads were analyzed by flow cytometry. Decrease in the reporter cell population (lower right quadrant) after PI treatment indicates non-viable mammalian cells. Shift of the population from the lower left quadrant to the upper left quadrant indicates non-viable yeast cells.

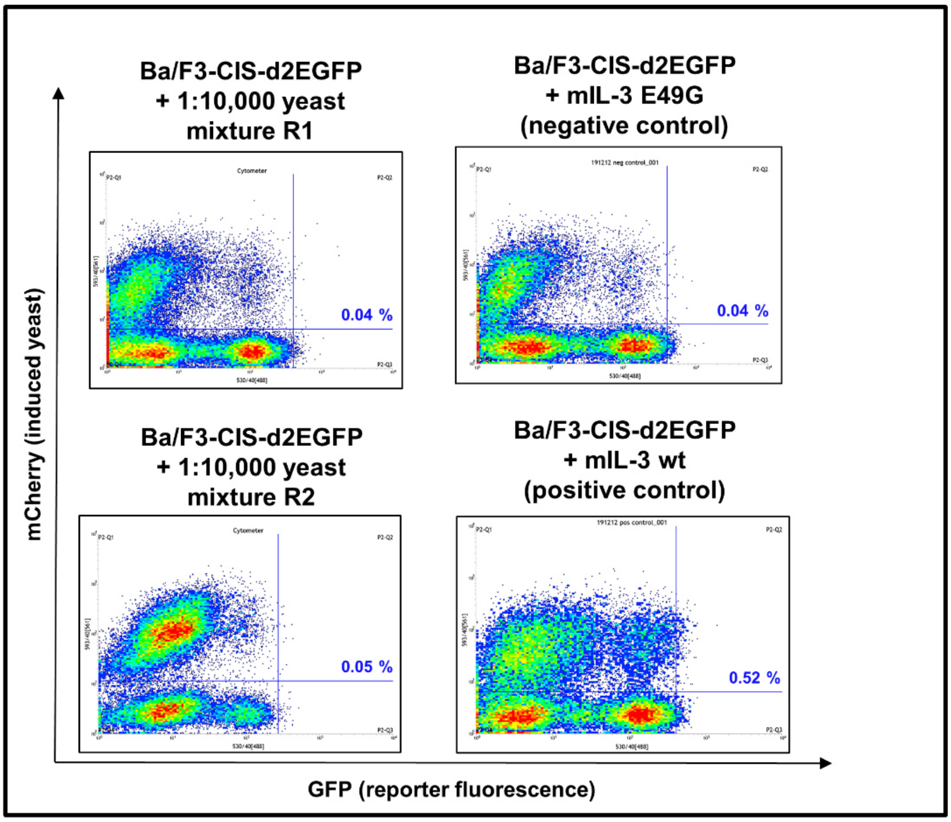

Figure S6. **FACS plots of first and second selection rounds of the 1:10,000 mIL-3 wt + mIL-3 E49G yeast mixture, co-encapsulated with mammalian reporter cells.** Positive control represents reporter cells co-encapsulated only with yeast secreting mIL-3 wt and negative control = reporter cells co-encapsulated with yeast cells secreting the non-functional E49G mIL-3 variant. High-stringency sorting gate (upper right quadrant P2-Q2) was used for selection of double positive agarose microbeads.

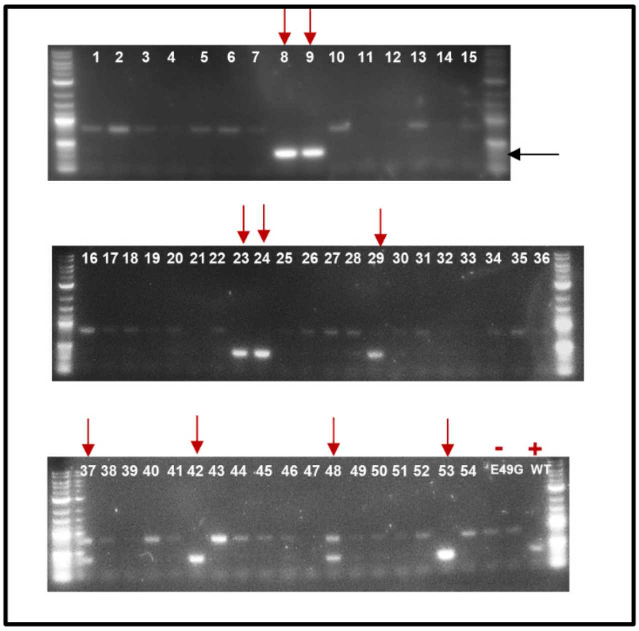

Figure S7. PCR screening of single clones after second functional screening selection round of 1:10,000 yeast mix (mIL3 wt + mIL-3 E49G). Band at about 400 bp (black arrow) represents a specific PCR amplicon for mIL-3 wt gene. Positive and negative controls represent mIL-3 wt and mIL-3 E49G single yeast clones, respectively, treated in the same way as the tested clones. Positive single clones are indicated with red arrows.

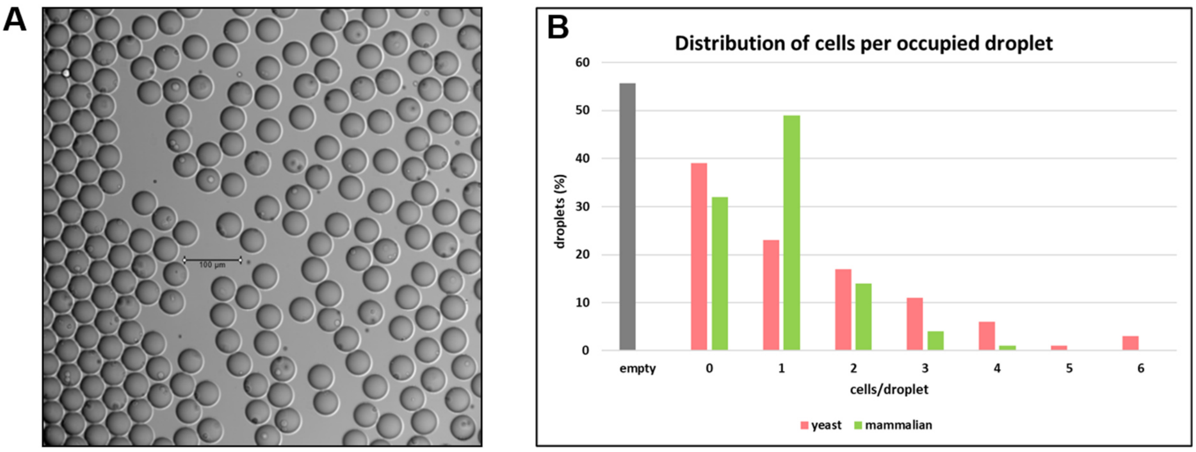

average droplet area	1530.58 μm^2
average droplet diameter	44.14 µm
average droplet volume	45.05 pL

Figure S8. Size and cell-distribution analysis of the generated agarose-containing droplets. (A) W/o emulsion was imaged on a microscopic slide without a cover slide in order to retain the spherical form of the droplets. Size analysis was performed by area measurement of 100 droplets with ImageJ using the 100 µm scale bar for calibration. Droplet diameter and volume were calculated based on the average droplet area. **(B)** Distribution of yeast and mammalian cells inside the droplets. 226 droplets were analyzed based on fluorescence microscopy images, 100 of the droplets contained cells and 126 were empty. Number of yeast and mammalian cells per corresponding droplet were estimated optically with help of the fluorescence signal of the cells (mCherry for yeast and GFP for mammalian reporter cells). Percentage of droplets with defined number of cells of a given cell type (0 to 6) per droplet was plotted.

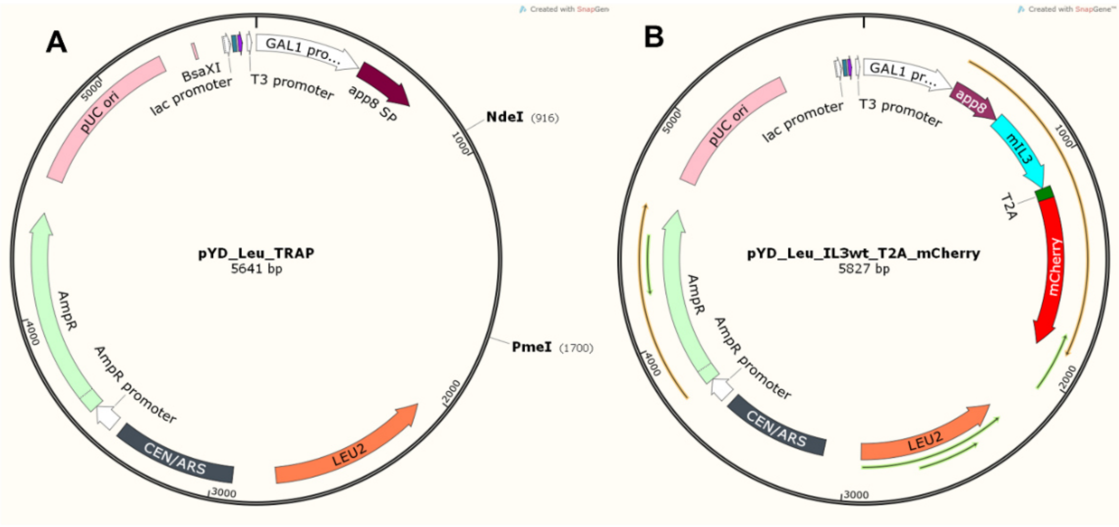

Figure S9. (A) Vector map of the initial pYD1 plasmid used for the generation of mIL-3 secretion plasmids. **(B)** Vector map of the *S. cerevisiae* mIL-3 wt-secretion plasmid, generated by gap repair cloning.

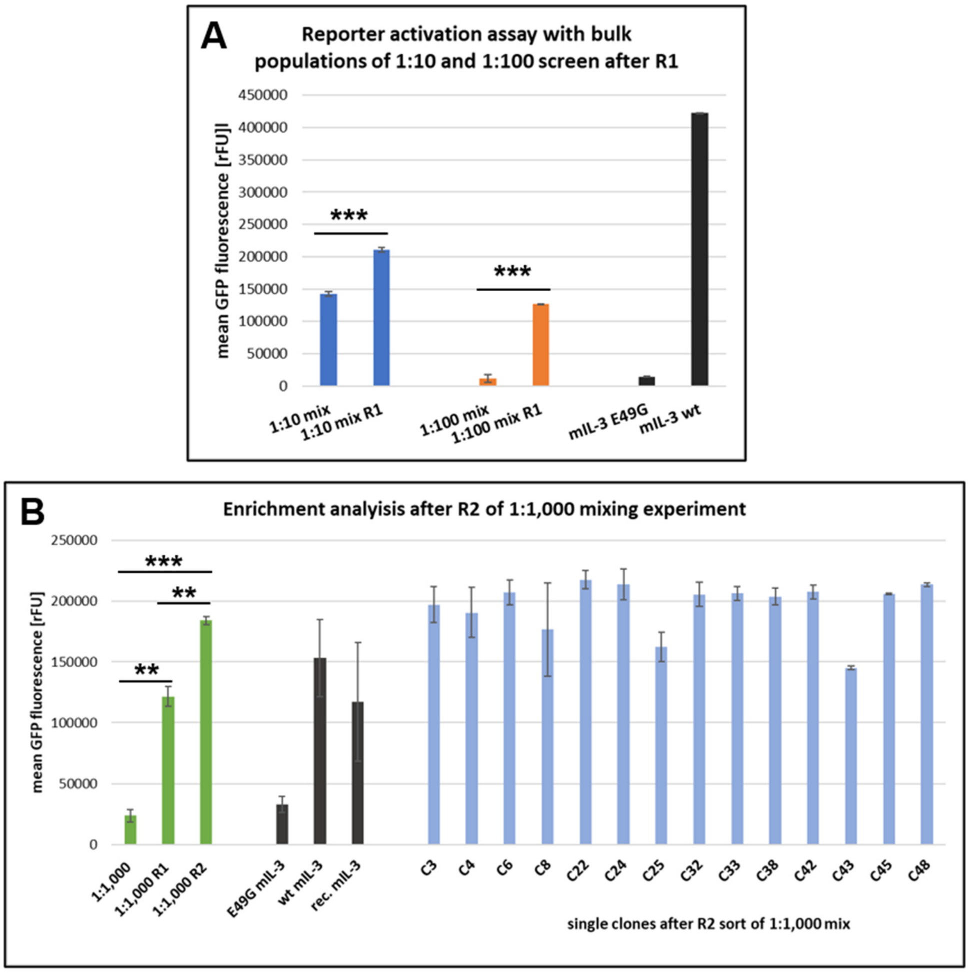

C

Ratio (wt : mutant)	Round 1		Round 2	
	wildtype clones	wildtype enrichment	wildtype clones	wildtype enrichment
**1:10**	3 of 13 (23.1 %)	2.3 times	×	×
**1:100**	2 of 13 (15.4 %)	15.4 times	×	×
**1:1,000**	2 of 52 (3.85 %)	38.5 times	14 of 54 (25.9 %)	259.3 times
**1:10,000**	not analyzed	not analyzed	9 of 54 (16.7 %)	1666.6 times

Figure S10. Additional mixing experiments with mIL-3 wt- mixed with an excess of mIL-3 E49G-secreting yeast cells. (A) Analysis of reporter cell activation by yeast supernatants from the 1:10 and 1:100 mixtures and corresponding populations after a single functional selection round. **(B)** Reporter cell activation assay with supernatant from the yeast mixture 1:1,000, as well as from bulk populations after R1 and R2 of functional selection. Single clones identified as mIL-3 wt after R2 *via* PCR were verified for secretion of functional mIL-3 cytokine. Statistical significance analysis was performed by One-way ANOVA and Turkey HSD post-hoc test. Relevant statistical significance between samples was depicted on the graph with asterisks. **(C)** Enrichment degree of mIL-3 wt-secreting yeast variant after functional selection of mIL-3 wt mixed with mIL-3 E49G mutant at different ratios.

Supplementary Tables

Table S1. Protein and DNA sequences of the multi-component cytokine expression construct for *S. cerevisiae*

	Amino acid sequence	DNA sequence
GAL1 promoter	X	acggattagaagccgccgagcgggtgacagccctccgaaggaagactc<br>tcctccgtgcgtcctcgtcttcaccggtcgcgttcctgaaacgcagatgtgc<br>ctcgcgccgcactgctccgaacaataaagattctacaatactagcttttatg<br>gttatgaagaggaaaaattggcagtaacctggccccacaaaccttcaaatg<br>aacgaatcaaattaacaaccataggatgataatgcgattagttttttagcctta<br>tttctggggtaattaatcagcgaagcgatgatttttgatctattaacagatatat<br>aaatgcaaaaactgcataaccactttaactaatactttcaacattttcggtttgt<br>attacttcttattcaaatgtaataaaagtatcaacaaaaaattgttaatatacct<br>ctatactttaacgtcaaggagaaaaaacc
app8 secretion signal	MRFPSIFTAVLFAASSALAA<br>PANTTTEDETAQIPAEAVID<br>YSDLEGDFDAAALPLSNSTN<br>NGLSSTNTTIASIAAKEEGV<br>QLDKR	atgagatttccttcaatttttactgcagttttattcgcagcatcctccgcattag<br>ctgctccagctaacactacaacagaagatgaaacggcacaaattccggct<br>gaagctgtcatcgattactcagatttagaaggggatttcgatgctgctgcttt<br>gccattgtccaacagcacaaataacgggttatcctccacaaatactactatt<br>gccagcattgctgctaaagaagaaggggtacaactcgataaaaga
mIL-3 wt mature (D33-C166)	DTHRLTRTLNCSSIVK**E**IIGK<br>LPEPELKTDDEGPSLRNKSF<br>RRVNLSKFVESQGEVDPEDR<br>YVIKSNLQKLNCCLPTSAND<br>SALPGVFIRDLDDFRKKLRF<br>YMVHLNDLETVLTSRPPQP<br>ASGSVSPNRGTVEC	gatacccaccgtttaaccagaacgttgaattgcagctctattgtcaaggaga<br>ttatagggaagctcccagaacctgaactcaaaactgatgatgaaggaccct<br>ctctgaggaataagagctttcggagagtaaacctgtccaaattcgtggaaa<br>gccaaggagaagtggatcctgaggacagatacgttatcaagtccaatcttc<br>agaaacttaactgttgcctgcctacatctgcgaatgactctgcgctgccagg<br>ggtcttcattcgagatctggatgactttcggaagaaactgagattctacatg<br>gtccaccttaacgatctggagacagtgctaacctctagaccacctcagccc<br>gcatctggctccgtctctcctaaccgtggaaccgtggaatgt
mIL-3 E49G	DTHRLTRTLNCSSIVK**G**IIGK<br>LPEPELKTDDEGPSLRNKSF<br>RRVNLSKFVESQGEVDPEDR<br>YVIKSNLQKLNCCLPTSAND<br>SALPGVFIRDLDDFRKKLRF<br>YMVHLNDLETVLTSRPPQP<br>ASGSVSPNRGTVEC	gatacccaccgtttaaccagaacgttgaattgcagctctattgtcaagggta<br>ttatagggaagctcccagaacctgaactcaaaactgatgatgaaggaccct<br>ctctgaggaataagagctttcggagagtaaacctgtccaaattcgtggaaa<br>gccaaggagaagtggatcctgaggacagatacgttatcaagtccaatcttc<br>agaaacttaactgttgcctgcctacatctgcgaatgactctgcgctgccagg<br>ggtcttcattcgagatctggatgactttcggaagaaactgagattctacatg<br>gtccaccttaacgatctggagacagtgctaacctctagaccacctcagccc<br>gcatctggctccgtctctcctaaccgtggaaccgtggaatgt
T2A	EGRGSLLTCGDVEENPGP	gagggccgcggcagcctgctgacctgcggcgacgtggaggaaaaccc<br>aggccca
mCherry	MCVSKGEEDNMAIIKEFMR<br>FKVHMEGSVNGHEFEIEGEG<br>EGRPYEGTQTAKLKVTKGG<br>PLPFAWDILSPQFMYGSKAY<br>VKHPADIPDYLKLSFPEGFK<br>WERVMNFEDGGVVTVTQD<br>SSLQDGEFIYKVKLRGTNFP<br>SDGPVMQKKTMGWEASSE<br>RMYPEDGALKGEIKQRLKL<br>KDGGHYDAEVKTTYKAKK<br>PVQLPGAYNVNIKLDITSHN<br>EDYTIVEQYERAEGRHSTGG<br>MDELYK	atgtgcgtgagcaagggcgaggaggataacatggccatcatcaaggagt<br>tcatgcgcttcaaggtgcacatggagggctccgtgaacggccacgagttc<br>gagatcgagggcgagggcgagggccgcccctacgagggcacccaga<br>ccgccaagctgaaggtgaccaagggtggcccctgcccttcgcctggga<br>catcctgtcccctcagttcatgtacggctccaaggcctacgtgaagcaccc<br>cgccgacatccccgactacttgaagctgtccttccccgagggcttcaagtg<br>ggagcgcgtgatgaacttcgaggacggcggcgtggtgaccgtgaccca<br>ggactcctccctgcaggacggcgagttcatctacaaggtgaagctgcgcg<br>gcaccaacttcccctccgacggccccgtaatgcagaagaagaccatggg<br>ctgggaggcctcctccgagagaatgtaccccgaggacggcgccctgaa<br>gggcgagatcaagcagaggctgaagctgaaggacggcggccactacg<br>acgctgaggtcaagaccacctacaaggccaagaagcccgtgcagctgc<br>ccggcgcctacaacgtcaacatcaagttggacatcacctcccacaacgag<br>gactacaccatcgtggaacagtacgaacgcgccgagggccgccactcc<br>accggcggcatggacgagctgtacaag

Table S2. Sequences for the specific primer used for PCR amplification for NGS analysis after functional screening and sorting of 1:10,000 mIL-3 wt and mIL-3-E49G yeast mixture. Primer consist of partial Illumina adapters, specific barcode (bold), and a mIL-3 gene homology sequence (lowercase). mIL-3 wt specific primer, used for PCR screen after second selection round.

	Primer	Sequence
1:10,000 mix	NGS IL3 Mix fwd	ACACTCTTTCCCTACACGACGCTCTTCCGATCT**TCTATATCGACACTGAGT**tgctgctaaagaagaagggg
	NGS IL3 Mix rev	GACTGGAGTTCAGACGTGTGCTCTTCCGATCT**ACTCAGTGTCGATATAGA**gataacgtatctgtcctcaggatc
1:10,000 mix R1	NGS IL3 MixR1 fwd	ACACTCTTTCCCTACACGACGCTCTTCCGATCT**TCTATATCGCACTCT**tgctgctaaagaagaagggg
	NGS IL3 MixR1 rev	GACTGGAGTTCAGACGTGTGCTCTTCCGATCT**AGAGTGCGATATAGA**gataacgtatctgtcctcaggatc
1:10,000 mix R2	NGS IL3 MixR2 fwd	ACACTCTTTCCCTACACGACGCTCTTCCGATCT**TCTATATCGCACGCG**tgctgctaaagaagaagggg
	NGS IL3 MixR2 rev	GACTGGAGTTCAGACGTGTGCTCTTCCGATCT**CGCGTGCGATATAGA**gataacgtatctgtcctcaggatc
mIL-3 wt specific	IL3 wt E23 fwd	CAGCTCTATTGTCAAGGAG
	IL3 rev	ACATTCCACGGTTCCACG

Table S3. Composition of agarose-containing encapsulation medium for generation of microdroplets by microfluidics.

Component	Stock	End concentration	Volume for 4 mL encapsulation medium
ultra-low gelling temp. agarose	3 % in medium mix	0.75 %	1 mL
FBS	100 %	20 %	800 µL
Opti-Prep™ density gradient medium	100 %	20 %	800 µL
Galactose	20 % in medium mix	2 %	400 µL
Penicillin-Streptomycin solution	10 000 units penicillin/mL 10 mg streptomycin/mL = 100 %	1 %	40 µL
Medium mix	50 % RPMI-1640 + 50 % DMEM-F12 Ham	X	1 mL

Table S4. Analysis of the statistical significance of the data set from Fig. 7A. One-way ANOVA, followed by Tukey HSD post-hoc test was performed on GraphPad Prism.

Tukey's multiple comparisons test	Mean Diff.	95.00 % CI of diff.	Significant?	Summary	Adjusted P Value
1:10,000 mix vs. R1	-25426	-46749 to -4104	Yes	*	0.017
1:10,000 mix vs. R2	-58266	-79588 to -36943	Yes	***	<0.001
1:10,000 mix vs. mIL-3 E49G	3419	-17904 to 24741	No	ns	0.993
1:10,000 mix vs. mIL-3 wt	-88245	-109567 to -66922	Yes	***	<0.001
1:10,000 mix vs. rec. mIL-3	-77372	-98694 to -56049	Yes	***	<0.001
R1 vs. R2	-32839	-54162 to -11517	Yes	**	0.002
R1 vs. mIL-3 E49G	28845	7523 to 50168	Yes	**	0.007
R1 vs. mIL-3 wt	-62819	-84141 to -41496	Yes	***	<0.001
R1 vs. rec. mIL-3	-51946	-73268 to -30623	Yes	***	<0.001
R2 vs. mIL-3 E49G	61685	40362 to 83007	Yes	***	<0.001
R2 vs. mIL-3 wt	-29979	-51302 to -8657	Yes	**	0.005
R2 vs. rec. mIL-3	-19106	-40429 to 2216	No	ns	0.089
mIL-3 E49G vs. mIL-3 wt	-91664	-112986 to -70341	Yes	***	<0.001
mIL-3 E49G vs. rec. mIL-3	-80791	-102113 to -59468	Yes	***	<0.001
mIL-3 wt vs. rec. mIL-3	10873	-10450 to 32195	No	ns	0.549

Table S5. Statistical significance analysis of the data set from Fig. 7B. One-way ANOVA, followed by Dunnett's multiple comparisons post-hoc test was performed on GraphPad Prism.

Dunnett's multiple comparisons test	Mean Diff.	95.00 % CI of diff.	Significant?	Summary	Adjusted P Value
mIL-3 E49G vs. C8	-369753	-473028 to -266478	Yes	***	<0.001
mIL-3 E49G vs. C9	-338403	-441678 to -235128	Yes	***	<0.001
mIL-3 E49G vs. C18	3312	-99963 to 106587	No	ns	>0.999
mIL-3 E49G vs. C23	-368856	-472131 to -265581	Yes	***	<0.001
mIL-3 E49G vs. C24	-400470	-503745 to -297195	Yes	***	<0.001
mIL-3 E49G vs. C29	-360940	-464215 to -257665	Yes	***	<0.001
mIL-3 E49G vs. C37	-381499	-484774 to -278224	Yes	***	<0.001
mIL-3 E49G vs. C42	-225308	-328583 to -122033	Yes	***	<0.001
mIL-3 E49G vs. C48	-317380	-420655 to -214105	Yes	***	<0.001
mIL-3 E49G vs. C49	7680	-95595 to 110955	No	ns	>0.999
mIL-3 E49G vs. C50	22578	-80697 to 125853	No	ns	0.996
mIL-3 E49G vs. C53	-273729	-377004 to -170454	Yes	***	<0.001
mIL-3 E49G vs. EBY100 wt	-531.1	-103806 to 102744	No	ns	>0.999
mIL-3 E49G vs. mIL-3 wt	-254918	-358193 to -151643	Yes	***	<0.001
mIL-3 E49G vs. rec. mIL-3	-256310	-359585 to -153035	Yes	***	<0.001
